# Primary hyperhidrosis prevalence and characteristics among medical students in Rio de Janeiro

**DOI:** 10.1371/journal.pone.0220664

**Published:** 2019-09-13

**Authors:** Maria Ribeiro Santos Morard, Ricardo Betanho Martins, Ana Carolina Lopes Ribeiro, Pedro Guimarães Rocha Lima, Beatriz dos Santos Carvalho, José Carlos Baldelim Santiago Junior

**Affiliations:** 1 UNIRIO—Universidade Federal do Estado do Rio De Janeiro, Rio de Janeiro, RJ, Brazil; 2 UNESA—Universidade Estácio de Sá, Rio de Janeiro, RJ, Brazil; 3 FMP–Faculdade de Medicina de Petrópolis, Petrópolis, RJ, Brazil; Faculty of Science, Ain Shams University (ASU), EGYPT

## Abstract

**Background:**

Hyperhidrosis is a pathological condition defined by excessive sweating beyond thermoregulatory physiological needs, which can cause substantial psychological impact and impairment of daily activities. Studies regarding its prevalence, however, are scarce and vary widely in their findings. The population of medical students is a particularly interesting subset for its recurring demand of physical contact during patient examination or procedures, and the potential for professional adversity. We aimed at furthering the comprehension of this disease prevalence and characteristics among medical students.

**Methods:**

Questionnaires inquiring about the presence and characteristics of Primary Hyperhidrosis (PH) were applied through either written or digital means to all eligible medical students enrolled in three Medical Schools in the State of Rio de Janeiro who agreed to take part in the study. Demographic data regarding gender, ethnicity, current age, weight and height was collected in addition to clinical data (sweat site, age of onset, familial history, severity and previous treatments). Severity was evaluated through the Hyperhidrosis Disease Severity Scale (HDSS) and a symptoms survey.

**Findings:**

Our response rate was roughly 1/3 of all eligible students (900/2700). PH prevalence was 20.56% (185/900). It was similar between men and women (23.08% and 19.41%, respectively) and strongly associated with family history of the disease (Prevalence Ratio of 4.27). Regarding ethnicity, of the total sample 73.78% (664/900) self-declared white, among which 19.28% (128/664) had PH. Mixed-race and other ethnicities encompassed 26.22% (236/900) of the sample, among which 24.15% (57/236) had PH. Most positive subjects (64.32%) presented associated forms of PH. Overall involvement of each site (both associated and isolated) was: 63.78% axillary, 50.81% palmar, 43.24% plantar, 20.54% craniofacial, 18.38% facial flushing and 2.16% gustatory sweating. Mean current age was 23.11(±4.04) years for PH patients, and age of onset was ≤18 years in 93.94% of cases. Regarding body mass index (BMI), 71.09% of PH patients had BMI<25kg/m^2^ and only 4.69% presented BMI≥30kg/m^2^, none ≥35kg/m^2^. Some degree of life quality impairment was reported by 89.20% of PH patients, and 23.89% had HDSS 3 or 4 (moderate to severe).

**Conclusions:**

PH prevalence among Rio de Janeiro medical students was 20.56%, similar between men and women, predominating associated presentations, axillary, palmar and plantar sites, strong familial history, age of onset before 18 years, and some degree of life impairment.

## Introduction

Hyperhidrosis is a pathological condition defined by excessive sweating beyond thermoregulatory physiological needs. Palmar, plantar and axillary regions are most often affected, isolate or in association, due to being the areas of highest density of eccrine glands, where the hypotonic secretion known as sweat is produced in greatest quantity. Other presentations exist but are rarer, such as Hexsel’s Hyperhidrosis, which affects the inguinal region. [1–3]

The eccrine glands are innervated by the sympathetic autonomic motor nervous system with hypothalamic input, via the thoracic sympathetic ganglia. Each sudomotor dermatome receives ipsilateral innervation from several adjacent segment levels: T1-T4 for the head, T2-T5 for the hand, T3-T6 for the armpits, T4-T12 for the trunk and T10-L4 for the leg and feet. [1, 2]

Primary hyperhidrosis (PH), in this context, is most often attributed to sympathetic hyperactivation. Histopathologic examination of sweat glands in affected individual show no signs of alteration. There is evidence of a genetic component in the disorder. [1, 4]

Secondary hyperhidrosis (SH) results of pregnancy, menopause, diabetes mellitus, obesity, fever, autonomic degenerative disorders, cerebral infarction, spinal cord injury, Harlequin syndrome, metabolic disorders (hyperthyroidism, pheochromocytoma), chronic infection, lymphomas, eccrine hamartomas, carcinoid syndrome, tuberculosis, AIDS and endocarditis. Drugs such as antidepressants, oral hypoglycemics, triptans, antipyretics, cholinergics and sympathomimetics can also be causes. It is, however, either less often localized than the primary form or asymmetric, as well as more often associated with additional clinical findings. [1–4]

PH prevalence, however, is poorly verified. [1] A research on PubMed with the keywords “Prevalence” and “Hyperhidrosis” on November 2018, returned 298 results, of which 15 were verified to be studies reporting Hyperhidrosis prevalence (Table 1).

**Table 1:** Prevalence studies.

Country	Author	Year	Sample	Prevalence	Reference
India	Muthusamy et al.	2016	492	38.00%^a^	[5]
Poland	Stefaniak et al.	2013	253	16.70%^a^^,^^b^	[6]
Germany	Augustin et al.	2013	14,336	16.30%^a^	[7]
Brazil	Lima et al.	2015	447	14.76%	[8]
China + Canada	Liu et al.	2016	2,028	14.50% and 12.30%	[9]
Japan	Fujimoto et al.	2013	5,807	12.76%	[10]
Brazil	Fenili et al.	2009	500	9.00%	[11]
Sweden	Shayesteh et al.	2016	1,353	5.50%	[12]
Brazil	Westphal et al.	2011	293	5.50%	[13]
USA	Doolittle et al.	2016	8,160	4.80%^a^	[14]
China	Tu et al.	2007	12,803	4.59%^c^	[15]
China	Li et al.	2007	33,000	4.36%	[16]
USA	Strutton et al.	2004	234,500	2.90%^a^	[17]
China	Lai et al.	2015	67,492	2.08%^c^	[18]
Brazil	Hasimoto et al.	2018	4,133	0.93%	[19]

^a^ Did not differentiate primary from secondary hyperhidrosis.

^b^ Prevalence value according to self-assessment rather than gravimetry for easier comparison.

^c^ Assessed only primary palmar hyperhidrosis.

The diagnosis is based on symptomatology, generally assessed through the Hyperhidrosis Disease Severity Scale (HDSS). Objective and quantitative methods of investigation exist but are seldom used in clinical practice. These include: gravimetry (the weight measurement of sweat collected on filter paper), dynamic quantitative sudometry (moisture measurement from dry gas) and Minor starch-iodine (a semi-quantitative test used for sweat mapping). [1, 4]

PH treatment revolves around three main pillars: clinical treatment, non-surgical procedures and surgical approach. Clinical treatment comprises topic use of antiperspirants, such as aluminum chloride and hydrochloride, which absorbs water and precipitates on glands orifices, and oral medication, such as anticholinergics (antagonizes eccrine sweat glands innervation. E.g.: Oxybutynin.). Non-surgical procedures are Iontophoresis, the formation of plugs on sweat channels through electrical current conduction, botulinic toxin A application, which impairs contraction of myoepithelial cells surrounding sweat glands on the dermis, and microwave ablation of sweat glands (thermolysis between deep dermis and hypodermis). [4]

Surgical approach revolves around thoracoscopic sympathectomy for treatment of palmar and axillary, and sometimes craniofacial PH. [20] Retroperitoneoscopic lumbar sympathectomy for plantar hyperhidrosis is still insipient and presents higher incidence of adverse effects than the thoracoscopic procedure, albeit with high levels of satisfaction (98%) reported by some authors. [21]

Despite presenting no mortality or risk for loss of organ or sense, there is evidence hyperhidrosis can cause considerable impairment of daily activities and psychological distress, interfering with work, leisure and social activities. Signs PH may affect a patient’s personality traits and interpersonal relations are found in increased association with alexithymia [22], fatigability and asthenia, self-forgetfulness and self-transcendence, as well as decreased association with purposefulness, resourcefulness, self-directedness. [23]. Prevalence of anxiety and depression among hyperhidrosis patients is nearly triple that of the population, proportional to disease severity. [24] Hyperhidrosis was characterized as one of the skin diseases that have major impact in quality of life, along with psoriasis, contact dermatitis, atopic dermatitis, urticaria, hair disorders, Hansen’s disease, scars and genital human papillomavirus disease. [12, 25]

With such concerns in mind, we sought to assess the prevalence, epidemiology and characteristics of PH among Medical Students in Rio de Janeiro and advise them about the disease and its treatment options.

## Methods

An observational, analytical cross-sectional study was designed to assess PH prevalence and performed after approval by the Ethics Committee (CAAE: 66911317.3.0000.5258, Report N° 2.431.144). The sample consisted of all enrolled students between the second halves of 2017 and 2018 in three Medical Schools in the State of Rio de Janeiro, Brazil: The Federal University of the State of Rio de Janeiro (UNIRIO), the Estácio de Sá University (UNESA) both in the City of Rio de Janeiro and the Faculty of Medicine of Petrópolis (FMP) in the City of Petrópolis.

Questionnaires investigating PH occurrence and characteristics, demographic data and personal history were applied by each center researchers to all eligible participants that could be contacted and agreed to take part in the study. Demographic data included gender, race, age, height and weight. Personal history included family history of PH, previous treatments for PH, satisfaction and adverse effects with previous treatments. PH characteristics were site, age of onset and severity (S1 and S2 Files).

Participants signed the Informed Consent and answered through either written or digital (Google Forms) means the form, which was accompanied by an excerpt detailing the research nature and purpose and suggesting literature on the disease.

Hyperhidrosis severity was evaluated with the Hyperhidrosis Disease Severity Scale (HDSS), the ubiquitous single straightforward question with four graded answers about the impact of symptomatology on daily life. It has been employed by almost every single PH prevalence study to this date and was recently translated and validated into Portuguese. [26]

Additionally, ten questions regarding impairment of specific activities, habits and feelings were asked to gauge which were most affected. These questions encompass embarrassment or impairment in daily activities, social events, sports, relationships, work environment, meetings and public speaking, as well as feelings of low self-esteem or of conveying a poor impression, and the impact on the choice of leisure activities, need for frequent baths and limiting of body movements.

On a second stage, students who reported PH on the questionnaire were invited for a consultation in person with a medical specialist involved with the research for diagnosis confirmation aimed primarily at ruling out secondary causes. Previously collected data was confirmed and further investigation of symptoms and history was performed encompassing symmetry, association with stress, presence of sweat dripping, presence of wet clothing, sweating in cold environments, improvement during sleep, absence of comorbidities and use of medications.

All collected data was downloaded (for digital responses) or manually imputed (for written responses) into an Excel sheet, where it was latter grouped and analyzed (S1 and S2 Tables). R Studio software was used to compare PH prevalence between groups by age, gender, ethnicity and presence of familial history. The tests for statistical significance used were the t test for continuous variable and chi square (χ^2^) test for categorical variables.

## Results

From the total population enrolled in all three Medical Schools, roughly a third (900/2700) responded the questionnaire. Our sample was overwhelmingly white and female and evenly distributed between centers.

PH prevalence among medical students in Rio de Janeiro was 20.56% (185/900) and, as expected, data showed no statistically significant difference between the three centers (χ_2_ = 1.7 p = 0.43). PH was slightly more prevalent among men than women, albeit with no statistical significance (χ_2_ = 1.02 p = 0.31) The detailed ethnic classification found very few individuals in some categories. We opted to compare white vs nonwhite groups that presented a prevalence ratio of 0.39 (χ_2_ = 53.1 p<0.0001). We found strong association between family history of PH and occurrence of the disease, with a prevalence ratio of 4.27 (presence of family history vs absence: χ_2 =_ 187.8 p<0.00000001) (Table 2). Mean age was 23.11 years for PH patients (standard deviation ±4.04) and 22.98 for non-PH respondents (ST ±4.01) (t = 0.39 p = 0.69).

**Table 2:** Population and PH prevalence demographics.

	Total, n (% of sample)	PH Prevalence	P value	PH Prevalence by Site					
				Axillary	Palmar	Plantar	Craniofacial	Facial Flushing	Gustatory Sweating
Total	900 (100%)	20.56% (185/900)							
UNIRIO	295	20.34% (60/295)							
UNESA	264	22.73% (60/264)							
FMP	341	19.06% (65/341)							
Gender									
Male	286 (31.78%)	23.08% (66/286)		14.69% (42/286)	11.19% (32/286)	10.14% (29/286)	4.90% (14/286)	4.55% (13/286)	0.70% (2/186)
Female	613 (68.11%)	19.41% (119/613)	0.31	12.89% (79/613)	10.44% (64/613)	8.81% (54/613)	4.24% (26/613)	3.75% (23/613)	0.33% (2/613)
Ethnicity									
White	664 (73.78%)	19.28% (128/664)		12.65% (84/664)	10.54% (70/664)	9.19% (61/664)	4.82% (32/664)	3.92% (26/664)	0.45% (3/664)
Mixed-race (W/B)	178 (19.78%)	25.28% (45/178)		17.98% (32/178)	11.24% (20/178)	10.11% (18/178)	3.93% (7/178)	5.06% (9/178)	0.56% (1/178)
Black	30 (3.33%)	13.33% (4/30)		10.00% (3/30)	10.00% (3/30)	6.67% (2/30)	0.00% (0/30)	0.00% (0/30)	0.00% (0/30)
Asian	10 (1.11%)	20% (2/10)		10.00% (1/10)	10.00% (1/10)	10.00% (1/10)	20.00% (2/10)	0.00% (0/10)	0.00% (0/10)
Indigenous	4 (0.44%)	0% (0/4)		0.00% (0/4)	0.00% (0/4)	0.00% (0/4)	0.00% (0/4)	25.00% (1/4)	0.00% (0/4)
Not informed	14 (1.56%)	42.86% (6/14)	p<10^−4^ *^a^	21.43% (3/14)	14.29% (2/14)	7.14% (1/14)	0.00% (0/14)	0.00% (0/14)	0.00% (0/14)
PH Family History									
Absent	728 (80.89%)	14.56% (106/728)		9.34% (68/728)	7.42% (54/728)	5.91% (43/728)	3.02% (22/728)	1.79% (13/728)	0.00% (0/728)
First-degree relative	101 (11.22%)	60.40% (61/101)		42.57% (43/101)	30.69% (31/101)	29.70% (30/101)	14.85% (15/101)	15.84% (16/101)	0.99% (1/101)
Second-degree relative	23 (2.56%)	69.57% (16/23)		43.48% (10/23)	43.48% (10/23)	39.13% (9/23)	13.04% (3/23)	21.74% (5/23)	13.04% (3/23)
Not informed	48 (5.33%)	4.17% (2/48)	p<10^−8^	4.17% (2/48)	2.08% (1/48)	2.08% (1/48)	2.08% (1/48)	4.17% (2/48)	0.00% (0/48)

*^a^ Comparison between “whites” and “nonwhites”.

The most commonly involved sites for PH were axillary, followed by palmar and then plantar. PH presentations encompassing multiple sites were more frequent than isolated involvement (64.32% vs 32.97%), and there were no isolated facial flushing or gustatory sweating cases. Regarding body mass index (BMI), 71.09% of PH patients had BMI<25kg/m^2^, 24,22% had BMI≥25kg/m^2^ and <30kg/m^2^, only 4.69% presented BMI≥30kg/m^2^, none ≥35kg/m^2^. From the six patients with BMI≥30kg/m^2^, three had involvement of a single site and three had involvement of two or more sites.

Some degree of life quality impairment (HDSS 2,3 or 4) was reported by 89.20% (165/185) of PH patients, and 23.89% (43/180) had HDSS 3 or 4 (moderate to severe).

All 10 people with PH onset after 18 years of age had axillary involvement (eight of them had isolated axillary involvement), and none of the eight who provided measurements for body mass index calculations were overweight (BMI≥25). Four had a first-degree relative with PH and six had no family history (Table 3).

**Table 3:** PH prevalence and characteristics by site.

	Involvement (% of all PH subjects)	Severity (% 3 or 4 on HDSS) ^a^	Age of onset (% ≤18 years) ^a^	BMI (% ≥25 kg/m^2^) ^a^	Average life impact—Degree of embarrassment or impairment: 1(none), 2 (mild), 3 (moderate), 4 (unbearable) ^b^									
Daily activities					Social events	Physical activities / sports	Work environment	Meeting and public speaking	Low self-esteem	Poor impression on people	Choice of leisure activities	Limitation to body movements	Increased frequency of baths
All presentations	100% (185/185)	23.9% (43/180)	93.94% (155/165)	28.91% (37/128)	2.10	2.12	2.01	1.61	1.91	1.75	2.13	1.54	1.98	2.05
Site (isolated or in association)														
Axillary	63.8% (118/185)	23.7% (28/118)	77.1% (91/118)	29.1% (23/79)	2.10	2.13	2.01	1.61	1.92	1.76	2.13	1.54	1.99	2.06
Palmar	50.8% (94/185)	24.5% (23/94)	98.9% (86/87)	27.0% (20/74)	2.10	2.13	2.01	1.61	1.91	1.75	2.13	1.54	1.98	2.05
Plantar	43.2% (80/185)	27.5% (22/80)	98.7% (75/76)	26.2% (17/65)	2.10	2.13	2.01	1.61	1.91	1.75	2.13	1.54	1.98	2.05
Craniofacial	20.5% (38/185)	21.1% (8/38)	97.0% (32/33)	34.6% (9/26)	2.12	2.14	2.03	1.62	1.92	1.77	2.13	1.56	2.01	2.06
Facial Flushing	18.4% (34/185)	26.5% (9/34)	96.8% (30/31)	26.3% (5/19)	2.11	2.14	2.01	1.60	1.90	1.78	2.13	1.54	2.01	2.05
Gustatory Sweating	2.2% (4/185)	0.0% (0/4)	100% (3/3)	-	2.17	2.17	2.03	1.65	1.95	1.78	2.14	1.56	1.99	2.12

Abbreviations: PH: Primary Hyperhidrosis; HDSS: Hyperhidrosis Disease Severity Scale; BMI: Body mass index.

^a^ Percentages were calculated based on total number of responses provided for each question.

^b^ Values expressed as mean of answers.

From the respondents claiming to have PH 12.43% (23/185) agreed to a consultation in person, of which 100% has the diagnosis confirmed by us. No SH subjects were incidentally detected on our study.

## Discussion

The published papers on the subject have reported hyperhidrosis prevalence on clinical diagnosis ranging between 0.93% and 16.70%. A research with the keywords “hyperhidrosis” and “medical students” on PubMed on March 1^st^, 2019 revealed 3 articles on the prevalence of PH among Medical Students. Ours is the third in Brazil and the fourth in the world, with the largest sample yet (900) (Table 1).

Our 20.56% PH prevalence finding was higher than those of the studies also among medical students in Aracaju, Brazil (14.76%) [8], and the self-reporting results in Poland (16.70%) [6], and much higher than those in Manaus, Brazil (5.5%) [13]. A study in India found an overall hyperhidrosis prevalence of 38% but included both medical and engineering students. [5]

Our PH prevalence was higher than Augustin et al. “focal hyperhidrosis” (16,30%), however, they did not differentiate primary and secondary presentations, as they considered only site distribution rather than multiple criteria such as age of onset, familial history, worsening with anxiety, improvement while sleeping and absence of known causes of SH. Their “focal hyperhidrosis” findings are thus not entirely comparable to our “PH” ones. [7] Additionally, Strutton (2.90%), Doolittle (4.8%) and Muthusamy (38%) did not differentiate primary from secondary hyperhidrosis. [5, 14, 17]

A Swedish study found a considerably lower prevalence of PH (5.5%), but a much larger prevalence of SH (14.8%) than us. [12] Similarly, a Brazilian study also diagnosed 29.4% of patients reporting excessive sweating as SH. [19] This suggests that differences in diagnostic criteria for primary or secondary presentations may be responsible for variability of PH prevalence rather than epidemiological differences.

We found similar prevalences of PH in men and women. This result is compatible with those also without statistical significance between genders prevalences, such as the Brazilian studies [8, 11, 13], a study in the USA [17] and a study in Sweden [12], but differs from those in Germany [7] and Japan (p<0.05) [10].

PH prevalence among nonwhite participants was higher compared to white ones. This contrasts with a Brazilian study that found no statistically significant difference between “whites”, “browns” and “blacks”. [8] It should be noted the Brazilian population is largely mixed-race and self-declared ethnicity may be variable. A study in Canada and China showed significant differences between ethnical groups regarding anatomical sites distribution, but not overall prevalence. [9]

Except for an American one [17], among all prevalence studies age of onset was overwhelmingly lower than 18–20 years [8, 14, 15, 18]. This is consistent with our findings.

According to the literature, axillary site is the most frequently affected, followed by palmar, plantar, inguinal and craniofacial. [4] All western studies regarding the primary form of hyperhidrosis found axillary, palmar and plantar sites to be more frequent than craniofacial. [8, 12, 13, 19]. A study in Japan found axillary and palmar PH to be the most frequent, but found craniofacial to be more frequent than plantar PH [10].

We found axillary site participation to be the most frequent, in accordance to the German and Swedish studies [7, 12] and in contrary to three Brazilian studies [8, 13, 19]. We found palmar PH to be slightly more prevalent than plantar PH, in accordance to studies in Brazil [13, 19], whereas the studies in Germany and Sweden found plantar hyperhidrosis to be slightly more prevalent than palmar hyperhidrosis. [7, 12]

Relative to family history, we found 45.9% of PH patients had a positive one. This is in consonance with the 30 to 50% in most studies. [8, 11, 13, 19]. The high level of association we found positive familial history and prevalence of the disease is in accordance with recent findings that suggest cholinergic and histological abnormalities (larger ganglia with thicker axonal myelin sheets) in sympathetic ganglia associated with genetic loci. [27]

Our study has shown 86.84% of PH participants reported some degree of impact on quality of life. These findings are greater than those by other authors. Other studies, however, might have underestimated them by not specifying examples of daily activities impairment in their questionnaires, possibly increasing recall bias. [8] An American study reported 75% of patients claiming negative impact on social life although without specifying whether the hyperhidrosis was primary. [14]

Few studies have evaluated subjective PH severity. We found that 20.54% (38/185) of students had severe symptoms (HDSS 3 or 4), a lower percentage than the 46.8% found in Japan. [10] It is also a much lower percentage than that found in the USA (70%) regarding overall (not necessarily primary) hyperhidrosis. [14]

We believe our chosen population has greater awareness and self-reporting of Hyperhidrosis, as well as other medical conditions, due to its technical background as medical students. It would, therefore, be more prone to characterizing abnormal sweat patterns as a disease rather than to dismiss them as physiological variation, compared to the general population. Evidence the ability to make this distinction is noteworthy is that an American study found 49% of hyperhidrosis patients had never discussed their condition with a healthcare provider, and of these, 60% claimed not thinking it was a medical condition and 47% believed there was no treatment available. [14]

The same technical knowledge decreases the chance of medical students misreporting secondary hyperhidrosis as primary. This includes the six positive respondents with BMI≥30kg/m^2^ (stage 1 obesity), who additionally had age of onset and focal involvement suggestive of primary etiology. Nevertheless, misreporting might be a possible source of bias for positive respondents who could not be interviewed in person.

Another possible explanation for the higher PH prevalence in our study is the proposition by some studies that the conditions ameliorates with aging. Since our population of medical students has a mean age of 23.11 years, it would exhibit a higher prevalence than studies that include higher age strata. Evidence for this is the fact a study in Germany found decreasing prevalence of focal sweating with increasing age [7], and that a study in Japan found 20% PH prevalence among adults 25–34 years of age, compared to 12.76% overall (5–64 years of age) [10]. Similarly, a study in Brazil found 11.8% PH prevalence among adults 18–30 years of age, compared to 9.0% overall (albeit with no statistical significance) [11], and an Indian study found 38% prevalence when assessing only college students 17 to 21 years-old. [5] Finally, an American study concluded hyperhidrosis prevalence to be the highest among 18–39 years old (8.8% compared to 4.8% overall). [14]

Given one of our study centers is situated in Petrópolis, a city on average 5.4°F (3°C) colder year-round than Rio de Janeiro, where the two other centers were located, the absence of statistically significant difference in PH prevalence between centers suggests no association between warmer climates and PH prevalence. This is in accordance with the absence of differences between Shanghai and Vancouver in another study. [9]

Prevalence studies might be prone to participation bias inflating the prevalence, since those with the disease (including PH) might be more likely to answer the study in hope their participation may lead to proper diagnosis and/or treatment. Our high response rate (900/2700), however, diminishes this effect, since even if all non-respondents were to be considered negative for the disease, our prevalence would still be above the literature minimum (in this hypothetical exercise it would be 6.85% (185/2700)).

## Supporting information

S1 File Questionnaire (Portuguese). (DOCX)

S2 File Questionnaire (English). (DOCX)

S1 Table Excel sheet with answers (Portuguese). Part of the data omitted to preserve confidentiality.
(XLSX)

S2 Table Excel sheet with answers (English). Part of the data omitted to preserve confidentiality.
(XLSX)
