# Supplementary material for: Primary hyperhidrosis prevalence and characteristics among medical students in Rio de Janeiro
Source: PLoS One. 2019 Sep 13;14(9):e0220664. doi: 10.1371/journal.pone.0220664 (PMC6744157; doi:10.1371/journal.pone.0220664)
Supplement: S1 File — (DOCX) [file pone.0220664.s001.docx]

Endereço de e-mail __________________________*

Você aceita o Termo de Consentimento Livre e Esclarecido? *

- Sim
- Não

Matrícula e iniciais do nome: *

_______________

Período no 2º Semestre de 2017 *

- 1º
- 2º
- 3º
- 4º
- 5º
- 6º
- 7º
- 8º
- 9º
- 10º
- 11º
- 12º

Sexo *

- Feminino
- Masculino
- Outro/Prefiro não informar

Cor *

- Branco
- Pardo
- Preto
- Indígena
- Amarelo
- Outro/Prefiro não informar

Idade ______

Possui história familiar de hiperidrose primária?

- Não
- Parente de 1º grau
- Parente de 2º grau
- Outro:

POSSUI HIPERIDROSE PRIMÁRIA (sudorese excessiva)? *

Caso NÃO, assinale a alternativa e siga para o final para enviar o formulário!

- SIM
- NÃO (siga para o fim do formulário)

Peso

_______

Altura

________

Qual a idade de início dos seus sintomas? _______

Qual a localização dos mesmos?

- Axilar
- Palmar
- Plantar
- Craniofacial
- Rubor facial
- Sudorese gustatória

Como você classificaria a gravidade de sua hiperidrose?

- Meu suor nunca é notável e não interfere nas minhas atividades diárias.
- Meu suor é tolerável, mas às vezes interfere nas minhas atividades diárias.
- Meu suor é dificilmente tolerável e frequentemente interfere nas minhas atividades diárias.
- Meu suor é intolerável e sempre interfere nas minhas atividades diárias.

O seu suor lhe causa constrangimento no dia-a-dia?

- Nada
- Um pouco
- Muito
- Muitíssimo

O quanto o seu suor lhe atrapalha ou causa constrangimento em eventos sociais?

- Nada
- Um pouco
- Muito
- Muitíssimo

O seu suor lhe incomoda ou causa constrangimento na prática de atividades físicas?

- Nada
- Um pouco
- Muito
- Muitíssimo

O quanto o seu suor afeta o seu trabalho ou prejudica o seu relacionamento com colegas?

- Nada
- Um pouco
- Muito
- Muitíssimo

O quanto seu suor lhe causa vergonha para falar em público ou em reuniões?

- Nada
- Um pouco
- Muito
- Muitíssimo

O quanto seu suor lhe causa a sensação de estar passando uma má impressão a alguém?

- Nada
- Um pouco
- Muito
- Muitíssimo

O quanto seu suor lhe causa um sentimento de baixa autoestima?

- Nada
- Um pouco
- Muito
- Muitíssimo

O quanto seu suor afeta suas escolhas de atividades de lazer? (por exemplos: viajar, dançar, praticar esportes)

- Nada
- Um pouco
- Muito
- Muitíssimo

O quanto seu suor faz com que você precise tomar banhos mais frequentes?

- Nada
- Um pouco
- Muito
- Muitíssimo

O quanto seu suor faz com que você limite seus movimentos corporais? (por exemplo: levantar os braços)

- Nada
- Um pouco
- Muito
- Muitíssimo

Você já realizou algum tratamento para a hiperidrose primária?

- Sim
- Não

Se sim, qual?

- Hexahidrato clorídrico de alumínio
- Iontoforese de água
- Injeção intradérmica de toxina botulínica
- Tratamento anticolinérgico com oxibutina ou glicopirrolato
- Clonidina
- Excisão de glândulas sudoríparas axilares
- Lipossucção axilar
- Simpatectomia toracoscópica
- Outro:

Após o tratamento, apresentou algum dos seguintes efeitos colaterais e complicações?

- Nervralgia intercostal
- Síndrome de Claude-Bernard-Horner
- Pneumotórax residual
- Infecção de ferida operatória
- Xerostomia (boca seca)
- Alteração cognitiva
- Outro:

Qual o grau de satisfação com o tratamento de 0-10 (sendo 0 completamente insatisfeito e 10 completamente satisfeito)?

- 0
- 1
- 2
- 3
- 4
- 5
- 6
- 7
- 8
- 9
- 10
