# Supplementary material for: Primary hyperhidrosis prevalence and characteristics among medical students in Rio de Janeiro
Source: PLoS One. 2019 Sep 13;14(9):e0220664. doi: 10.1371/journal.pone.0220664 (PMC6744157; doi:10.1371/journal.pone.0220664)
Supplement: S2 File — (DOCX) [file pone.0220664.s002.docx]

E-mail __________________________*

Do you accept the Informed Consent Agreement? *

- Yes
- No

Student ID Number and Name Initials: *

_______________

Academic Term/Semester in the 2^nd^ Half of 2017*

- 1^st^
- 2^nd^
- 3^rd^
- 4^th^
- 5^th^
- 6^th^
- 7^th^
- 8^th^
- 9^th^
- 10^th^
- 11^th^
- 12^th^

Gender *

- Male
- Female
- Other/I would prefer not to inform

Ethnicity*

- White
- Mixed-race (W/B)
- Black
- Indigenous
- Asian
- Other/I would prefer not to inform

Age ______

Do you have family history of Primary Hyperhidrosis?

- No
- First-degree relative
- Second-degree relative
- Other:

DO YOU HAVE PRIMARY HYPERHIDROSIS (excessive sweating)? *

If NOT, mark ‘No” and proceed to the end of the form!

- YES
- NO (proceed to the end)

Weight

_______

Height

________

What was the age of onset of your symptoms? _______

Where are your symptoms located?

- Axillary
- Palmar
- Plantar
- Craniofacial
- Facial Flushing
- Gustatory Sweating

How would you rate the severity of your hyperhidrosis?

- My sweating is never noticeable and never interferes with my daily activities.
- My sweating is tolerable but sometimes interferes with my daily activities.
- My sweating is barely tolerable and frequently interferes with my daily activities.
- My sweating is intolerable and always interferes with my daily activities.

Do you feel embarrassed by your sweating during daily activities?

- Not at all
- A little/mildly
- A lot/moderately
- Extremely/unbearably

Do you feel embarrassed or hindered by your sweating in social events?

- Not at all
- A little/mildly
- A lot/moderately
- Extremely/unbearably

Do you feel embarrassed or distressed by your sweating during physical activities/sports?

- Not at all
- A little/mildly
- A lot/moderately
- Extremely/unbearably

Are your work and professional relationships impaired by your sweating?

- Not at all
- A little/mildly
- A lot/moderately
- Extremely/unbearably

Do you feel embarrassed by your sweating in meetings and when speaking in public?

- Not at all
- A little/mildly
- A lot/moderately
- Extremely/unbearably

Do you feel you are conveying a poor impression of yourself to others because of your sweating?

- Not at all
- A little/mildly
- A lot/moderately
- Extremely/unbearably

Do you have low self-esteem because of your sweating?

- Not at all
- A little/mildly
- A lot/moderately
- Extremely/unbearably

Does your sweating influence your choice of leisure activities (e.g.: travelling, dancing, playing sports)?

- Not at all
- A little/mildly
- A lot/moderately
- Extremely/unbearably

Have you increased the frequency of your baths because of your sweating?

- Not at all
- A little/mildly
- A lot/moderately
- Extremely/unbearably

Does your sweating cause limitation to body movements (e.g.: raising your arms)?

- Not at all
- A little/mildly
- A lot/moderately
- Extremely/unbearably

Have you ever attempted any treatment for primary hyperhidrosis?

- Yes
- No

If yes, which?

- Aluminum chloride hexahydrate
- Tap water iontophoresis
- Intradermic botulinum toxin injection
- Anticholinergics (Oxybutynin or Glycopyrrolate)
- Clonidine
- Axillary sweat glands excision
- Axillary liposuction
- Thoracoscopic sympathectomy
- Other:

Did you present any of the following adverse effects and complications following treatment?

- Intercostal neuralgia
- Claude-Bernard-Horner Syndrome
- Residual pneumothorax
- Surgical site infection
- Xerostomia (dry mouth)
- Cognitive fluctuation
- Other:

How satisfied were you with the treatment from 0 (completely unsatisfied) to 10 (completely satisfied)?

- 1
- 2
- 3
- 4
- 5
- 6
- 7
- 8
- 9
- 10
